# Death in the Pantaloon

**Published:** 1872-10

**Authors:** 


### DEATH IN THE PANTALOON.

THE dying general said, "It will be a strange thing, if, after having escaped unhurt in so many hotly contested battle-fields, I should lose my life through a pair of old army trowsers." He did die shortly after. On an emergency he exchanged a pair of pantaloons made of stout woolen for another pair very much worn. It was merely a mistake; there did not seem to be time to rectify it; but in a few hours the weather became much colder; he was not in a position to make it possible to put on a warmer garment; he got chilled through and through. In two or three days pneumonia developed itself, and thus passed away one of the bravest of the brave, not yet an old man, by any means. Many a reader can remember a life lost by an injudicious change of clothing.

Never change to lighter clothing after dressing in the morning; nor on starting on a journey; nor on going to church; nor on visiting a public assembly; because in either of these situations there are almost insuperable obstacles to making an alteration in the dress, in case of children, or a sudden change to colder weather. Rachel, the great tragedienne, who received the homage of kings and conquerors, left the New York Academy of Music one winter's night for Boston; the weather became colder; she had not access to her wardrobe; the fires were allowed to burn down; when she arrived at her journey's end she was most thoroughly chilled; a bad cold, a shroud, the grave, finished her history. Two continents mourned a premature death. But the coffers of the Czar of all the Russias could not purchase exemption from the consequences of neglecting those precautions which are necessary to health and life itself, not even in the case of so great an actor.

